# Evaluation of a novel non-invasive preimplantation genetic screening approach

**DOI:** 10.1371/journal.pone.0197262

**Published:** 2018-05-10

**Authors:** Valeriy Kuznyetsov, Svetlana Madjunkova, Ran Antes, Rina Abramov, Gelareh Motamedi, Zenon Ibarrientos, Clifford Librach

**Affiliations:** 1 CReATe Fertility Center, Toronto, Canada; 2 Department of Obstetrics and Gynecology, Faculty of Medicine, University of Toronto, Toronto, ON, Canada; 3 Department of Physiology, University of Toronto, 1 King's College Circle, Toronto, ON Canada, Canada; 4 Institute of Medical Sciences, University of Toronto, 1 King's College Circle, Toronto, ON Canada, Canada; 5 Department of Gynecology, Women’s College Hospital Toronto, ON, Canada; Western University, CANADA

## Abstract

**Objective:**

To assess whether embryonic DNA isolated from blastocyst culture conditioned medium (BCCM) combined with blastocoel fluid (BF) could be used for blastocyst stage non-invasive preimplantation genetic testing for chromosomal aneuploidy (non-invasive preimplantation genetic screening, NIPGS).

**Patients:**

47 embryos from 35 patients undergoing IVF.

**Interventions:**

DNA analysis of combined BCCM plus BF in comparison with trophectoderm (TE) biopsy and/or whole blastocyst (WB)using next generation sequencing (NGS).

**Results:**

Embryonic DNA was successfully amplified in 47/47 NIPGS samples (28 frozen-thawed and 19 fresh culture samples) ranging from 6.3 to 44.0 ng/μl. For frozen-thawed embryos, the concordance rate for whole chromosome copy number per sample was equivalent between NIPGS vs. TE biopsy, NIPGS vs. WB and TE vs. WB samples taken from the same embryo was 87.5%; 96.4% and 91.7% respectively (P>0.05), and the rate of concordance per single chromosome was 99.3%, 99.7% and 99.7%, respectively (P>0.05). In fresh cases (Day 4 to Day 5/6 culture), the concordance rate for whole chromosome copy number per sample between NIPGS vs. TE samples taken from the same embryo was 100%, and the rate of concordance per single chromosome was 98.2% (P>0.05).

**Conclusions:**

A combination of BCCM and BF contains sufficient embryonic DNA for whole genome amplification and accurate aneuploidy screening. Our findings suggest that aneuploidy screening using BCCM combined with BF could potentially serve as a novel NIPGS approach for use in human IVF.

## Introduction

Chromosomal abnormalities lead to implantation failure, early pregnancy loss or severe chromosomal diseases such as Down’s and Patau syndromes [1, 2]. It is estimated that more than 50% of miscarriages are due to aneuploidy [3]. Recent studies suggest that transfer of euploid embryos assessed through such techniques as array comparative genomic hybridization (aCGH), next-generation sequencing (NGS) or qPCR based comprehensive chromosomal screening of trophectoderm (TE) biopsies of blastocysts, significantly improves implantation and ongoing pregnancy rates, and decreases miscarriage rates [4,5].

One of the major limitations of PGS is the presence of chromosomal mosaicism within the developing embryo. Embryonic mosaicism poses significant risk for misdiagnosis as the cells biopsied may not reflect the chromosomal status of the entire embryo. Although there appears to be a high concordance rate between TE cells and ICM cells [6], in selected blastocysts a TE biopsy may not always represent the remainder of the TE or the inner cell mass (ICM) [5, 7, 8]. When NGS is used for chromosomal analysis, detection of mosaicism is increased up to 30% compared to aCGH [9, 10]. Greco et al [11] recently reported a series of chromosomally mosaic embryos that developed into healthy euploid newborns, however miscarriage rates were increased.

TE biopsy is technically challenging and invasive [12, 13].TE biopsy samples typically contain about 4 to 6 cells. Increasing the number of biopsied cells might improve accuracy, but is likely to reduce implantation rate [14].Non-invasive preimplantation genetic screening (NIPGS) offers an alternative non-invasive approach which avoids embryo biopsy [13].

RecentlyDNA, whose extracellular origin is still under investigation, has been found in human embryonic blastocoel fluid (BF)[15, 16].Therefore, BF may represent a source of embryonic genetic material that is routinely discarded during the vitrification process to prevent ice crystal formation and improve embryo survival following cryopreservation[17]. Compared to TE biopsy, BF aspiration using an ICSI pipette is a less invasive approach to obtain embryonic genetic material, less technically challenging for an embryologist trained to perform ICSI, and more cost effective [18, 19]. A few studies have shown that BF DNA represents to as good an extent (or possibly even better), the blastocyst chromosomal composition, when compared with biopsied TE cells [18, 19], and has the potential to be used for preimplantation aneuploidy screening [15, 20, 21]. However clinical utilization of this approach requires improvements in the efficiency of the DNA analysis process.

Recently, cell-free embryonic DNA has also been found in blastocyst culture conditioned medium (BCCM) [21, 22]. The first promising results on using BCCM for preimplantation genetic diagnosis [21, 22] and preimplantation genetic screening [23, 24] were only recently published.

However, to our knowledge, there are no published studies to evaluate the accuracy of BCCM combined with BF in comparison to TE biopsy and WB testing from the same embryo, using NGS. Herein, we will present our data on this novel approach.

## Materials and methods

### Ethics approval

This study was approved by the University of Toronto Research Ethics Board (IRB no. 30251). Written informed consent was obtained from all study participants.

### Sample collection

For this study, as a proof of principle, we used 28 cryopreserved human blastocysts donated for research. Most of the embryos donated for research (85.7%) were embryos which had already been diagnosed as abnormal by preimplantation genetic testing for aneuploidy (PGT-A). In addition, BCCM and BF from 19 fresh cultured embryos was collected prior to TE biopsy and cryopreservation. The 28 donated frozen embryos were derived from a total of 26 patients aged 25 to 45 years (mean 37.5 +/-5.8 years). The 19 fresh embryos were from 9 patients, aged 35 to 42 years (mean 38.9+/-3.2 years).

### NIPGS on donated frozen embryos

**Fig 1:**
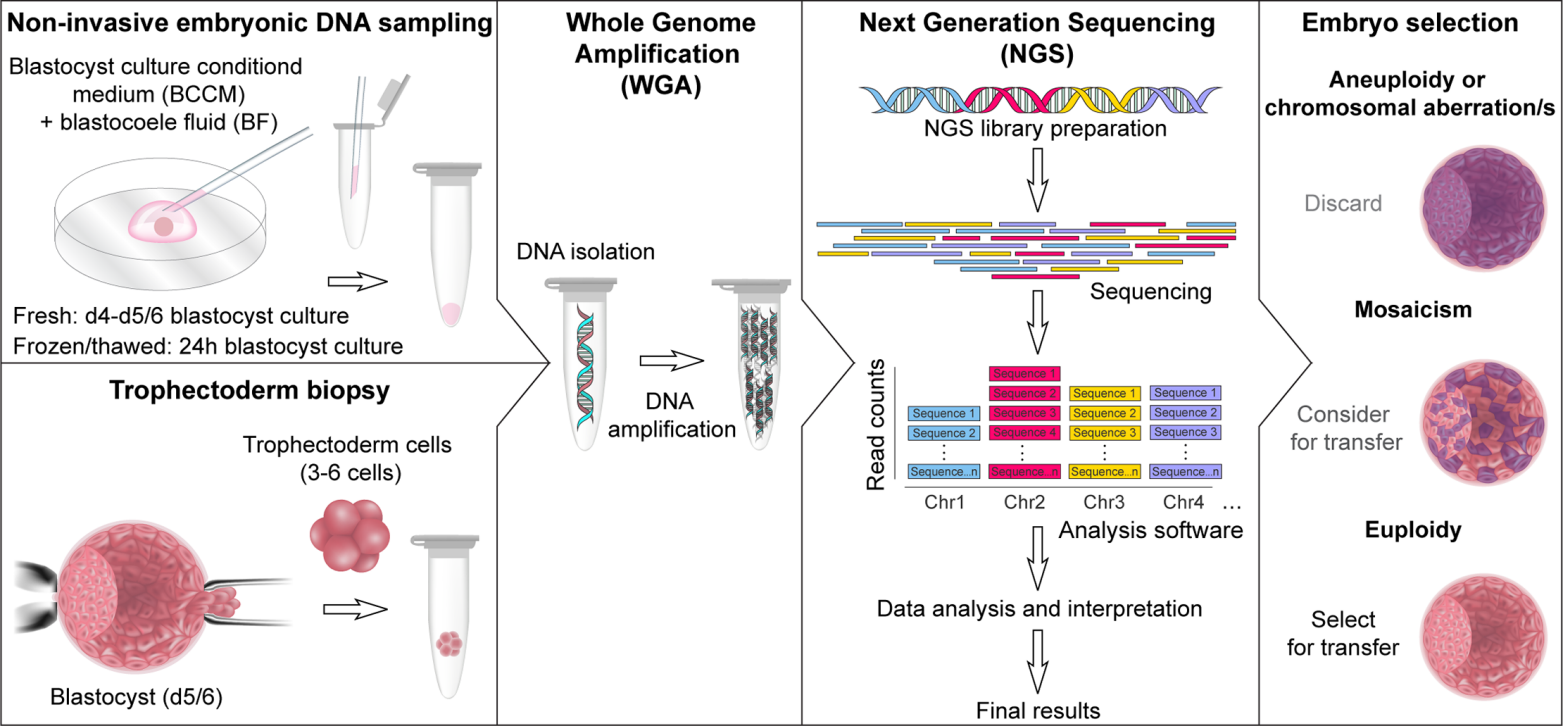
Non-invasive and invasive preimplantation genetic testing workflow.

The workflow for processing of donated frozen embryos is illustrated in Fig 1 and detailed as follows.

Mature (metaphase II) oocytes were fertilized by intracytoplasmic sperm injection (ICSI). After ICSI, the fertilized oocytes were cultured individually from Day 1 (D1) to D5/6 in Sage 1-Step medium with serum protein supplement (Origio, Denmark) under oil, in 25-μL droplets.

Twenty-four of the 28 frozen embryos (85.7%) underwent TE biopsy before cryopreservation. This involved laser-assisted biopsy of 4–6 trophectoderm (TE) cells, which was performed in Global “total” w/Hepes and protein medium (LifeGlobal, Belgium) under oil, as described previously [25]. All were frozen by vitrification after laser collapse of the blastocoel cavity, as per standard procedures [17]. PGT-A was performed using standard NGS protocol for VeriSeq PGS kit (Illumina, CA) after SurePlex whole genome amplification of biopsied TE cells. Data analysis and visualization was done using BlueFuse Multi Software (Illumina, CA).

For this part of the study (proof of principle), the 28 embryos were warmed and then placed in separate 25-μL droplets of Global HP medium with HSA (LifeGlobal, Belgium), and cultured under oil for 24 hours (Note, during protocol optimization, a shorter incubation time of 8 hours was attempted with 6 additional embryos; results for these were suboptimal and are discussed below, but not included in the analysis). After culture all blastocysts subsequently re-expanded during the 24 hours. They were then collapsed by laser, allowing the BF to extrude from the blastocoel cavity and mix with the BCCM. After removal of each embryo, the combined fluid was collected and frozen at -80°C for NIPGS analysis, and the whole embryo was processed for PGS, as described below. Four blastocysts in the cohort had not been biopsied prior to the original cryopreservation because they were observed to be either 1PN or 3PN embryos. The remainder of the embryo (WB) were processed separately for PGS, as described below. Culture medium alone incubated in the same micro droplet dish was used as a negative control.

### NIPGS on freshly cultured embryos

We performed NIPGS on the combined BCCM and BF from 19 freshly cultured embryos from a total of 9 patients. All patients consented to donate their BCCM and BF for research, rather than discard it as waste. The workflow for these embryos is illustrated in Fig 1 and detailed as follows. Each embryo was transferred on day 4 of culture to fresh Global HP medium with HSA (Life Global, Belgium), and continued to be cultured under oil until they reached the blastocyst stage (on day 5 or 6). Upon reaching a fully expanded blastocyst, each blastocyst underwent laser assisted trophectoderm biopsy. This was followed by laser collapse, which allowed the BF to mix with the BCCM. The embryo was then transferred to cryopreservation medium and frozen by vitrification, as per standard procedures [17].

After removal of the embryo, the combined BCCM and BF NIPGS samples were collected and frozen at -80C until tested. WGA and chromosome screening of these NIPGS samples was then carried out subsequently, as described below.

### Whole genomic amplification (WGA) and chromosome screening

DNA from all samples was amplified using SurePlex kit (BlueGnome) according to the manufactures instructions and quantified by Qubit3.0 Fluorimeter (Thermo Fisher Scientific). Amplified DNA was assessed for whole and segmental chromosome aneuploidy screening with a previously validated VeriSeq™ PGS kit on the MiSeq system (Illumina) in the CReATe Fertility Centre Genetics Laboratory [26].

### Assessment of the chromosomal content

**Fig 2:**
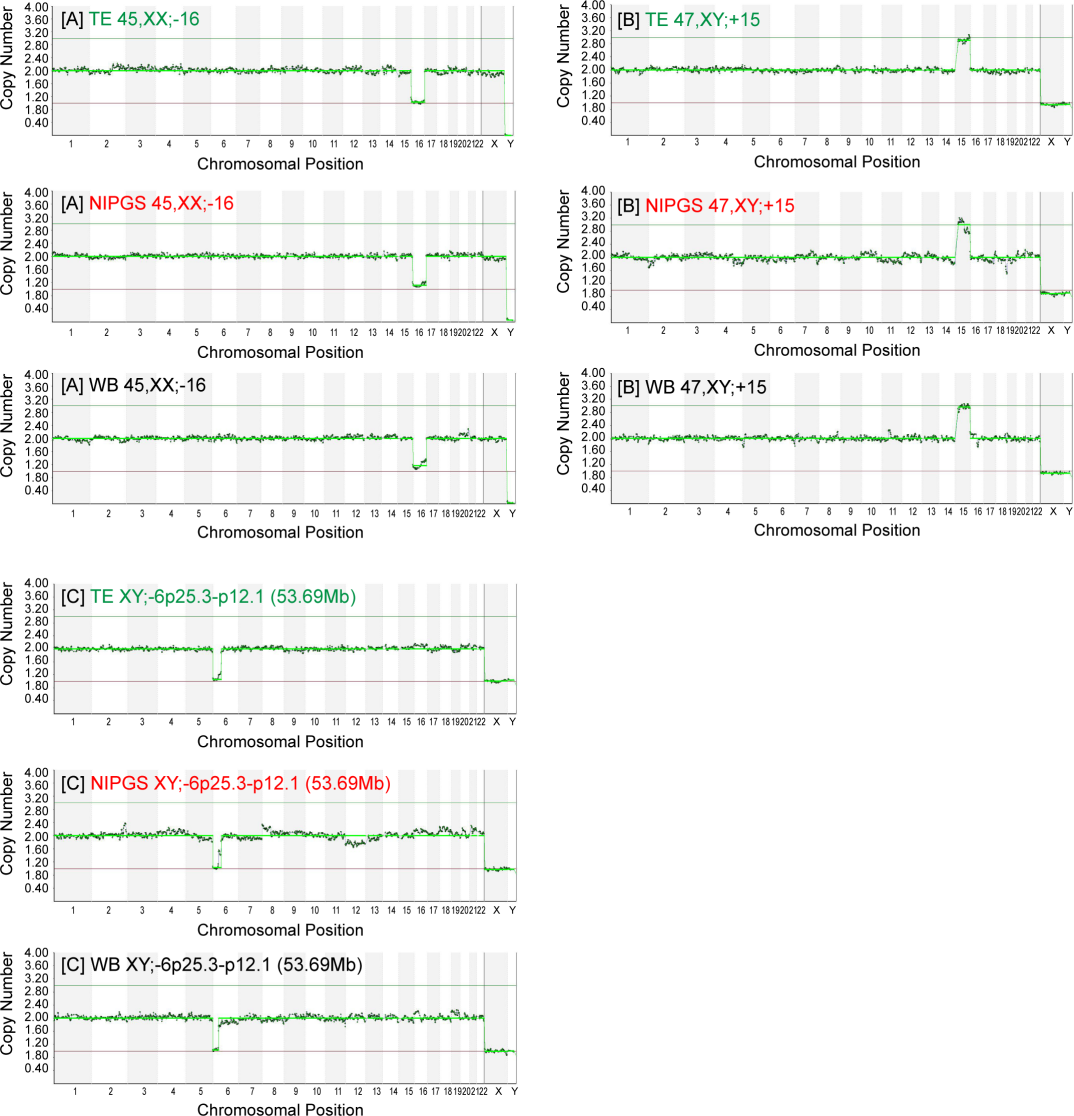
Three examples comparing TE biopsy, NIPGS and WB from the same vitrified-thawed embryo. Fig 2A, 2B and 2C show consistent results between TE biopsy, NIPGS and WB samples. Size of segmental loss detected in 2C was smaller in the WB sample compared with the TE and NIPGS samples (37.15 Mb vs. 53.69 Mb).

NIPGS, TE and WB samples were processed using the same protocol as described above, assessed for ploidy status (euploid vs. aneuploid) using BlueFuse Multi software (Illumina, CA) and, in the case of aneuploid embryos, the type and size of detected chromosomal aberrations was indicated (Fig 2). Results were considered as fully concordant only when ploidy and chromosomal aberrations fully matched between sample types. Partial concordance was indicated when only some of chromosomal aberrations between samples were in agreement. Results where samples showed no concordance with regards to chromosomal composition were classified as discordant.

### Statistical analysis

For statistical analysis, whole blastocyst (WB) results (in the frozen donated group only), were regarded as the reference "gold-standard". Analyses of concordance of the WB result compared to TE and NIPGS was performed using McNemar’s test. Where applicable, statistical analysis was performed using two-tailed chi-square testing or Fisher exact testing, and results were considered significant when P<0.05.

## Results

### Protocol optimization

During protocol optimization, we attempted to isolate and amplify DNA from six blastocysts cultured post-thaw for only 8 hours, but the DNA quantity in combined BCCM and BF samples was not sufficient to produce conclusive NGS results post WGA. Analysis of these six short-term culture NIPGS samples resulted in low DNA yields post WGA (range 4.4–7.88 ng/μl), 5/6 (83.3%) samples resulted in chaotic DNA signals, and 1 sample exhibited noisy profile not meeting necessary quality control scores for interpretation. These results are not included in the analysis below.

### NIPGS on donated frozen embryos

The amplification rate for embryonic DNA from NIPGS (combined BCCM and BF) samples collected from embryos cultured for 24 hours post thaw was 100% (28/28). DNA concentration in each sample after WGA was found to range between 25.0 and 54.0 ng/μl for TE biopsy, from 10.5 to 44.0 ng/μl for NIPGS and from 21.3 to 65.6 ng/μl for WB. Respective blank medium negative controls associated with each sample that underwent WGA showed no amplification in all cases. The concordance rate for whole chromosome copy number per sample between NIPGS vs. TE, NIPGS vs. WB and TE vs. WB samples taken from the same embryo was 87.5%; 96.4% and 91.7% accordingly (p>0.05), and the rate of concordance per single chromosome was 99.3%, 99.7% and 99.7%, respectively (P>0.05) (Fig 2, Tables 1 and 2). NIPGS accurately identified gender for all samples.

**Table 1:** Summary of NGS results from NIPGS, TE and WB samples obtained from the same blastocyst.

Sample Number	TE biopsy*	NIPGS	WB
**1**	XX; +18	XX; +18	XX; +18
**2**	XX; +20	XX; +20	XX; +20
**3**	XX; -10, +21q21.2–22.3 (21.05Mb)	XX; normal	XX; -10
**4**	XY; +4, +9q	XY; +4	XY; +4
**5**	NA	XX; normal	XX; normal
**6**	XX; +22	XX; +22	XX; +22
**7**	NA	XX; normal	XX; normal
**8**	NA	68, XXY; -21	68, XXY; -21
**9**	NA	XX; +22	XX; +22
**10**	XY; +12	XY; +5q (70%)	XY; normal
**11**	XX; +22	XX; normal	XX; normal
**12**	XY; +19	XY; +19	XY; +19
**13**	XX; -22	XX; -22	XX; -22
**14**	XY; -18; -21	XY; -18; -21	XY; -18; -21
**15**	XY; +22	XY; +22; mosaic -8 (40%)	XY; +22
**16**	XY; +12q21.1–24.33 (62.08Mb)	XY; +12q21.1–24.33 (62.08Mb)	XY; normal
**17**	XY; +15, +16	XY; +15, +16	XY; +15, +16
**18**	XY; -16	XY; -16	XY; -16
**19**	XX; -16	XX; -16	XX; -16
**20**	XX; +17	XX; +17	XX; +17
**21**	XY; +15	XY; +15	XY; +15
**22**	XY; +11, mosaic -21 (50%)	XY; +11	XY; +11
**23**	XY; +19	XY; +19, mosaic -5 (30%)	XY; +19
**24**	XX; +16	XX; +7, +16	XX; +16
**25**	XY; -11, +12pter-q14 (64.65Mb)	XY;-11, +12pter-q14 (63.48Mb)	XY;-11, +12pter-q14 (63.48Mb)
**26**	XX; +9, -17	XX; +9, -17	XX; +9, -17
**27**	XY; -4, +22	XY; -4, +22	XY; -4, +22
**28**	XY; -6p 25.3–12.1 (53.69Mb)	XY; -6p 25.3–12.1 (53.69Mb)	XY; -6p 25.3–21`.2 (37.15Mb)

NA = not available

TE = Trophectoderm

NIPGS = Noninvasive preimplantation genetic screening

WB = Whole blastocyst

* Clinical PGT-A results from TE biopsy perform prior initial embryo vitrification.

**Table 2:** Concordance of blastocyst ploidy and karyotype per sample and per chromosome assessed by NIPGS vs TE vs WB.

Analysis of chromosomal aberrations									
Type of samples		*Full concordance*		*Partial concordance*		*Discordant*		*Total concordance*	
		*Blastocyst*	*Per Chromosome*	*Blastocyst*	*Per Chromosome*	*Blastocyst*	*Per Chromosome*	*Blastocyst*	*Per Chromosome*
		*(N/%)*	*(N/%)*	*(N/%)*	*(N/%)*	*(N/%)*	*(N/%)*	*(N)*	*(N/%)*
**NIPGS vs. TE biopsy**	**Embryo ploidy**	21/24 (87.5)						24	
	**WCN**	20 (83.3)	480/480 (100)	1 (4.2)	23/24 (95.8)	3 (12.5)	69/72 (95.8)	24	572/576 (99.3)
	All aberrations*	16 (66.7)	384/384 (100)	5 (20.8)	110/120 (91.7)	3 (12.5)	69/72 (95.8)	24	563/576 (97.8)
**NIPGS vs. WB**	**Embryo ploidy**	27/28 (96.4)^a^						28	
	**WCN**	26 (92.8)	624/624(100)	1 (3.6)	23/24 (95.8)	1 (3.6)	23/24 (95.8)	28	670/672 (99.7)^b^
	All aberrations*	22 (78.5)	528/528 (100)	5 (17.9)	115/120 (95.8)	1 (3.6)	23/24 (95.8)	28	666/672 (99.1)
**TE biopsy vs. WB**	**Embryo ploidy**	22/24 (91.7)^a^						24	
	**WCN**	22 (91.7)	528/528 (100)	0	0	2 (8.3)	46/48 (95.8)	24	574/576 (99.7)^b^
	All aberrations*	18 (75.0)*	432/432 (100)	4 (16.7)	428/432 (99.1)	2 (8.3)	46/48 (95.8)	24	570/576 (98.9)

WCN = *whole chromosome copy number*

*Chromosomal aberrations include: whole chromosome aneuploidy, segmental aneuploidy and/or mosaicism

^a^McNemar’s test: The two-tailed P value equals 0.4881.Same superscript in the column indicates the difference are not statistically significant

^b^Fisher exact testing: Same superscript in the column indicates the difference are not statistically significant (P>0.05)

As indicated in Table 2, ploidy concordance between each of the three sample types (BCCM, TE biopsy and WB) was 87.5–96.4%.Of the NIPGS samples with single whole chromosome aneuploidy, 11 were trisomies, and 5 were monosomies.

This method was also capable of detecting unbalanced translocations. In one patient, a balanced translocation carrier (46,XX, t(11:12)(q24.2;q15)), NIPGS was able to detect chromosomal aberrations resulting from the above mentionedtranslocation, which was also detected in TE and WB samples (Table 1, sample number 25).

### NIPGS on freshly cultured embryos

The amplification rate for embryonic DNA from NIPGS (combined BCCM and BF) samples collected from embryos cultured for 24–48 hours in fresh media after day 4 was 100% (19/19). DNA concentration in each sample after WGA was found to range between 25–46 ng/μl for TE biopsy and from 6.3 to 36.0 ng/μl for NIPGS. Respective blank medium negative controls associated with each sample that underwent WGA showed no amplification in all cases. The mean amount of NIPGS sample DNA obtained from the fresh blastocyst was lower that from vitrified-warmed blastocysts (15.2 ± 2.7 ng/μl vs. 21.7 ± 5.2 ng/μl, respectively), however not statistically different.

**Fig 3:**
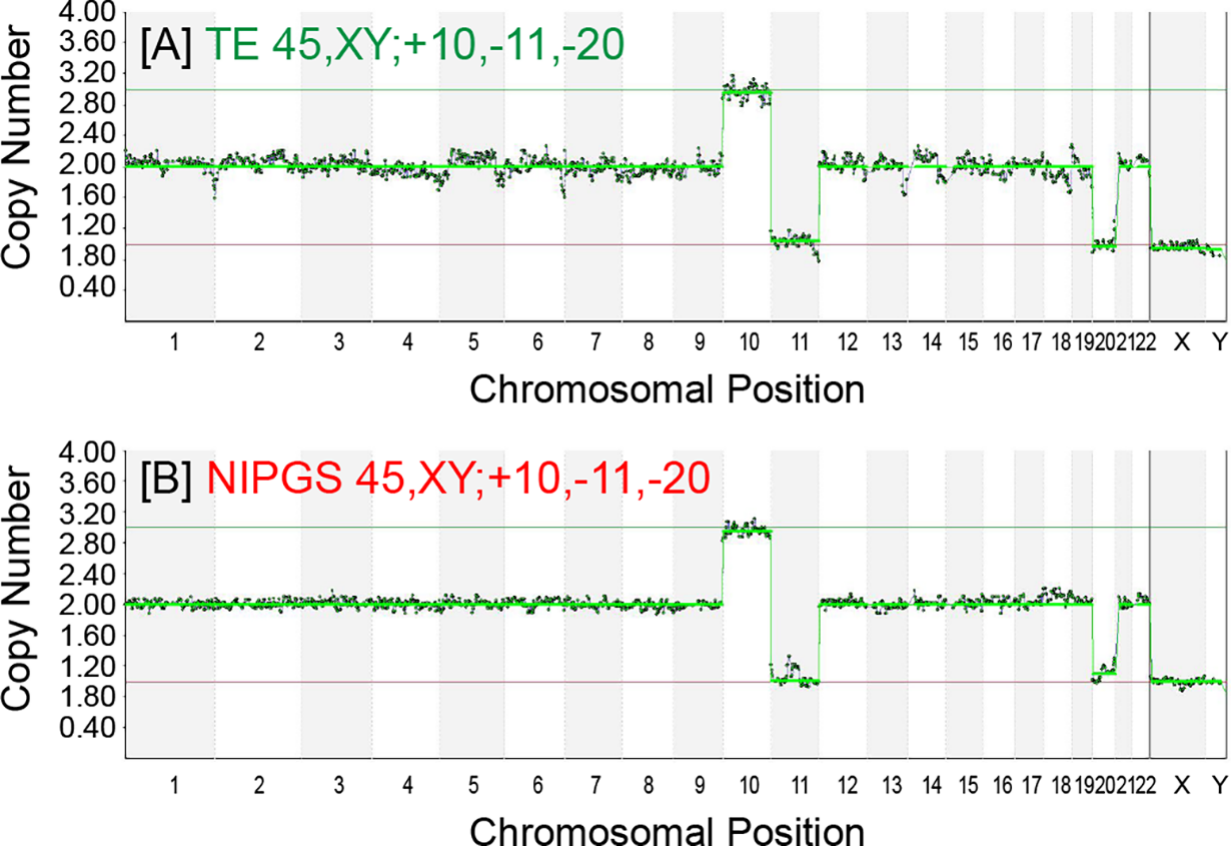
One example comparing TE biopsy and NIPGS from the same freshly cultured embryo. Fig 3A and 3B show consistent results between TE biopsy and NIPGS samples for this embryo.

The concordance rate for whole chromosome copy number per sample between NIPGS vs. TE biopsy samples taken from the same embryo was 100%, and the rate of concordance per single chromosome was 98.2%, (Fig 3, Tables 3 and 4).

**Table 3:** Summary of NGS results from NIPGS and TE samples obtained from the same blastocyst in fresh cases.

Sample Number	TE biopsy	NIPGS	WGA DNA concentration in NIPGS
**1**	XY; normal	XY; mosaic -8 (70%)	16.9 ng/μl
**2**	XY; mosaic loss: (-4q31.3-q35.2, 32.9Mb, 20%)	XY; mosaic gain: (+4q 22.1-q35.2, 94.3Mb, 30%)	30.0 ng/μl
**3**	XY; -5, -13	XY; -5, -13	26.0 ng/μl
**4**	XY; mosaic -9 (30%)	XY; normal	22.5 ng/μl
**5**	XX; +13, +19, -21	XX; +13, +19, -21	23.7 ng/μl
**6**	XY; +10, -11, -20	XY; +10, -11, -20	22.6 ng/μl
**7**	XY; mosaic +6q22.1-q.25.2 (38.5Mb, 45%), mosaic -15 (50%)	XY; mosaic -8 (50%)	36.0 ng/μl
**8**	XX; normal	XX; normal	25.4 ng/μl
**9**	XX; normal	XX; normal	6.3 ng/μl
**10**	XX; -18q12.2-q23 (43.34Mb) -12q11.2–12.1 (18.89Mb)	XX; -18q12.2-q23 (43.34Mb) -12q11.2–12.1 (18.89Mb)	9.3 ng/μl
**11**	XY; mosaic -4q (118.6Mb, 20%)	XY; mosaic +13q21.1–31.3 (34.84Mb, 60%)	11.7 ng/μl
**12**	XX; normal	XX; normal	17.1 ng/μl
**13**	XX; normal	XX; normal	11.4 ng/μl
**14**	XX; mosaic loss: (-3p.26.3 –p25.2, 12Mb, 35%)	XX; normal	21.0 ng/μl
**15**	XX; -14	XX; -14	14.0 ng/μl
**16**	XX; normal	XX; normal	6.44 ng/μl
**17**	XY; normal	XY; normal	14.4 ng/μl
**18**	XX; -15	XX; -15	13.1 ng/μl
**19**	XY; +16, +21`	XY; +16, +21	22.9 ng/μl

TE = Trophectoderm

NIPGS = Non-invasive preimplantation genetic screening

**Table 4:** Concordance of blastocyst ploidy and chromosomal aberrations per sample and per chromosome assessed by NIPGS vs. TE biopsy.

Analysis of chromosomal aberrations									
Type of samples		*Full concordance*		*Partial concordance*		*Discordant*		*Total concordance*	
		*Blastocyst (N/%)*	*Per Chromosome (N/%)*	*Blastocyst (N/%)*	*Per Chromosome (N/%)*	*Blastocyst (N/%)*	*Per Chromosome (N/%)*	*Blastocyst (N/%)*	*Per Chromosome (N/%)*
**NIPGS vs. TE biopsy**	Embryo ploidy	19/19 (100)^a^						19 (100)	
	WCN	19 (100)	456/456 (100)	0	0	0	0	19 (100)	456/456 (100)^a^
	All aberrations*	14 (73.7)	336/336 (100)	5 (26.3)	112/120 (95.8)	0	0	19 (100)	448/456 (98.2)

WCN = *whole chromosome copy number*

*Chromosomal aberrations include: whole chromosome aneuploidy, segmental aneuploidy and/or mosaicism

^a^Fisher exact testing: Same superscript in the column indicates the difference are not statistically significant (P>0)

## Discussion

To avoid the disadvantages of performing an invasive embryo biopsy with its associated risk, and the requirement for an embryologist skilled in performing embryo biopsy, an accurate non-invasive method to obtain representative DNA would be ideal for diagnostic PGD/PGS testing in IVF centers.

Over the last decade, non-invasive prenatal testing for fetal aneuploidy and monogenic disorders has been achieved [27, 28]. Apoptosis appears to be the main mechanism controlling the release of cell-free fetal DNA from the placenta into the maternal circulation [29]. Apoptosis and release of cell-free DNA also appears to take place in the human embryo when cultured *in vitro* [23]. Therefore, analysis of cell-free DNA released from embryos may be useful for the analogous development of non-invasive preimplantation genetic screening.

For non-invasive embryonic genetic screening using BCCM to become reliable, several steps in the process must be optimized. These include improving DNA collection methods, DNA amplification, and downstream techniques for analysis.

In this study, we demonstrated that cell-free embryonic DNA is released into BF and BCCM from frozen Day 5/6 blastocysts post-thaw after culture for 24 hours, as revealed by 100% successful amplification of DNA from these NIPGS samples. This proof of principal portion of the study is also unique in that we not only compared NIPGS samples to TE biopsy samples, but we also compared both of them to the WB analysis as a gold standard control.

We chose to analyze the combination of BF and BCCM vitrified day 5/6 blastocysts after warming and culture for 24 hours for two reasons. Firstly, we hypothesized that combining both together as one sample would increase the amount and quality of cell-free genomic DNA obtained. Secondly, we also hypothesized that the collection of BCCM after 24h in culture, rather than longer, would minimize cell-free DNA degradation. In contrast, BCCM collected from an embryo cultured in monophasic media (grown in the same media droplet from day 1 to Day 5/6 (blastocyst) [30] may, on one hand, lead to a higher quantity of DNA released into the media, but on the other hand, may result in increased DNA degradation over the 5–6 days of culturing.

The novelty of our approach is that allows for completely non-invasive collection of cell free embryonic DNA released into BCCM and BF without adding additional procedures to the clinical IVF laboratory workflow. Previous reports analyzing BF for NIPGS utilized ICSI pipettes to collect BF [15, 16, 18, 19, 20]. Our technique does not require this additional step. In addition, to our knowledge, no other group has utilized a combination of both BCCM and BF. The combination of these two fluids increases the total amount of cell free DNA in the final sample. Having more DNA theoretically would reduce the risk of having an amount of DNA that is below the detection limit for the technique. This should reduce amplification failures and increase reliability of this test in a clinical setting.

For the genetic analysis of samples with a low DNA concentration, the WGA step is critically important. In our study, we used the SurePlex™ (PicoPlex) DNA amplification system that reproducibly amplifies DNA from single or small numbers of cells. Using a different amplification system, Huang et al [31] showed that NGS using the MALBAC™ kit (Yikon Genomics) provided concordant chromosomal results when compared with aCGH and SNP array in blastocysts with chromosomal abnormalities. Deleye et al [32] compared these two amplification systems and showed that SurePlex amplification led to more uniformity across the genome, allowing for better copy number aberration (CNAs) detection of deletions, duplications, and unbalanced translocations larger than 10Mb, with less false positives compared to the MALBAC system. In our experiments, most of the amplified NIPGS samples gave lower yields of genomic DNA when compared with TE and WB samples.

The accuracy and reliability of DNA screening methods are also critical in the development of preimplantation genetic testing [3, 26, 33]. For this study we used NGS as our approach for preimplantation genetic testing using the VeriSeq PGS kit (Illumina). NGS has rapidly become the standard method used around the world with higher robustness, accuracy and increased sensitivity for chromosomal aberrations and mosaicism detection than aCGH [3, 26, 33].

Our study demonstrates that genomic DNA isolated from a combination of BCCM and BF can be successfully isolated, amplified, and assessed. We showed that the amplification rates of embryonic DNA from these NIPGS samples was high (47/47, 100%) and compares favourably with the generally accepted 1–2% failed amplification rate that results from TE biopsy samples [34]. Our results show superior amplification rates of 100% over previously reported amplification rates from BCCM or BF alone, which range from 76.5% to 94.4% [15, 16, 18, 19, 21]. We obtained PGT-A results using NGS for 100% of NIPGS samples and a relatively high concordance rate between NIPGS vs. TE biopsy, NIPGS vs. WB and TE biopsy vs. WB samples (87.5% vs. 96.4% vs. 91.7% per sample, p>0.1, and 99.3%, 99.7% and 99.7% per chromosome, p>0.1) respectively (Tables 1 and 2). Liu WQ et al [35] used the NGS approach for aneuploidy detection and showed ploidy concordance of 64%, while Xu J et al. [23] showed sensitivity and specificity rates of 88.2% and 84%, both using MALBAC amplification system and BCCM as a source of cfDNA in comparison to TE-biopsy. Lane M et al. [36] and Vera-Rodriguez M et al. [37] used SurePlex amplification of BCCM and reported aneuploidy concordance rates of around 65%. They indicated that this relatively low concordance rate was likely caused by maternal contamination. During development of this approach we explored and evaluated the efficacy of WGA (SurePlex) of cfDNA in BF aspirated by ICSI pipette (S1 Fig) and had an amplification efficacy of only 60% (n = 15) and aCGH PGT-A results in 30% of the cases, which is similar to the results reported in above mentioned studies (S1 Fig). Furthermore, in the optimization process we evaluated the amplification efficacy using SurePlex WGA kit and NGS analysis for PGT-A detection using the VeriSeq kit when only BCCM was used as well as different approaches of combining BCCM and BF (with and without zona breaching at day 4, aspiration with ICSI pipette or laser collapse) as shown in S1 Table. Amplification efficacy and downstream utility of the combined BCCM+BF samples were better which directed our efforts into development of the novel approach that we present here.

In our study not all aneuploid TE samples were concordant with NIPGS (Table 1, samples 3, 10 and 11) and with WB (Table 1, samples 10 and 11) samples. We speculate that these discrepancies are most likely due to embryo mosaicism as it was shown by Vera-Rodriguez M et al [36]. However, there are some other possible explanations for this discordance: a) maternal (cumulus) contamination of media samples [36, 37, 38]; and b) DNA collected from the NIPGS samples, especially the BF component, may be more representative of the ICM chromosomal complement. In our clinical laboratory, we have established a protocol to control for maternal contamination using fluorescently labelled short tandem repeat (STR) marker analysis of embryonic DNA together with a source of maternal DNA. However, in the presented study, we did not have cumulus cells or another source of maternal DNA to assess maternal contamination. Segmental abnormalities detected in the TE samples (Table 1, samples 16 and 28) were also identified in the corresponding NIPGS samples (Table 1, samples 16 and 28), but in only one of the WB samples (Table 1, sample 28). Interestingly, NIPGS samples could potentially be used for NGS-based translocation detection as in sample 25 (Table 1) where the mother was a carrier of a balanced translocation involving chromosomes 11 and 12 [t(11:12)(q24.2;q15)].Results from sample number 8 (3PN blastocysts) reveal that NIPGS samples may also be useful for the detection of triploid embryos by NGS.

One limitation of using frozen thawed embryos in our proof of principle experiments to test our hypothesis, is that it is possible that for some embryos the rate of apoptosis seen after a vitrified embryo is thawed, is higher than when an embryo is freshly cultured. This could potentially increase the amount of cell free DNA present and allow for easier and more accurate detection after amplification. In addition, this model is not practical for NIPGS use in clinical IVF practice.

Therefore, we performed NIPGS on 19 freshly cultured embryos, after collection of combined BCCM and BF prior to vitrification. We transferred these embryos into fresh medium on day 4 of cultured them until blastocyst formation. Our results (Tables 3 and 4 and Fig 2), show that these NIPGS samples contained sufficient DNA for WGA and testing. Although the mean amount of DNA obtained from the amplification was lower than that obtained from vitrified-warmed blastocysts the difference was not statistically significant. We hypothesize that this difference results from 3 potential causes: a)DNA from apoptotic cells may add to the total cell free DNA in the case of vitrified-warmed blastocysts, b)the number of cells in an embryo at the day 4 morula stage is significantly lower than in a fully formed blastocyst, likely leading to lower amounts of cell free DNA released during the pre-blastulation to expanded blastocyst transition period [37]; and c) the total time the blastocoele cavity is present to accumulate cell free DNA is usually less in the fresh cases.

Recently, cell-free nuclear DNA observed in blastocoel fluid and in embryo culture conditioned medium, was used to characterize several genes (*HBB*, *MTHFR*) of the embryos [21, 22].

Although the mechanism of DNA secretion is not known, it is most likely that the embryo genomic DNA in the BCCM and BF originates from apoptotic cells within the growing embryo [23, 39]. Previously studies using differential labelling of the trophectoderm and inner cell mass nuclei with DNA-specific fluorochromes have led to the identification of a wave of apoptosis in the inner cell mass as the blastocyst expands [39]. Apoptosis is characterized by fragmentation of DNA into nucleosome-sized fragments of approximately 180 base pairs, whereas necrosis results in more random fragmentation and longer sized fragments [34]. By sequencing, Zhang et al [20] identified two fragmentation patterns in BF DNA; a dominant population of 160–220 bp (major peak at 169 bp) and a minor population of 300–400 bp. The dominant population of BF-DNA fragments had a length that was similar to the length of circulating cffDNA that originates from apoptotic placental trophoblast cells [28, 29].

The limitations of NIPGS include several considerations: 1) Because cell-free DNA in the BF and spent embryo culture medium contains very short DNA fragments and likely originates from apoptotic cells [21–23, 20], one concern is whether the DNA in the BCCM medium represents the full chromosomal complement of the embryo. However, if the origin of this DNA is predominantly from the inner cell mass, NIPGS samples might even be more representative of the future fetus [34]; 2) Depending on how long the DNA is present in the sample collected, there is a risk of degradation. Based on our data from a replacement of fresh medium on day 4 of in vitro culture, it appears that 24–48 hours gives a good enough yield to achieve consistent DNA amplification. This would require the use of sequential medium. 3) There is a potential risk of maternal contamination in the collected medium from residual cumulus cells. Prevention of this is critical. In our laboratory, we carefully remove and wash off cumulus-corona radiata cells before ICSI. Washing and replacing culture medium on Day 4 may also decrease the likelihood of maternal contamination due to residual cumulus cells; 4) Were commended that NIPGS be performed in conjunction with ICSI to avoid possible supernumerary sperm attached to the zona pellucida and thus avoid the risk of paternal contamination; and 5) For those IVF laboratories that routinely culture multiple embryos together in droplets, in order to use this NIPGS technique, the embryos must be removed, washed, and then transferred to individual droplets on Day 4 of culture. Since we culture embryos individually in our laboratory, we have not attempted NIPGS on embryos derived from coculture conditions.

## Conclusions

To the best of our knowledge, this is the first report describing NIPGS of blastocysts using combined BCCM and BF samples. We have demonstrated a 100% amplification rate for these NIPGS samples and a high concordance between BCCM, TE biopsy and WB (control) samples for whole chromosome copy number. From our encouraging preliminary results, we feel that non-invasive preimplantation genetic testing (NIPGT) for chromosomal abnormalities assessment has the potential to be an accurate and reliable option for preimplantation genetic testing of human embryos. However more research and technical refinement is still needed to perfect NIPGS so that it can be used in routine clinical practice.

## Supporting information

S1 Fig **A. Blastocoel fluid (BF) collection.** Aspiration of BF from a blastocyst using an ICSI pipette: gentle aspiration allows the blastocoel cavity to collapse. Approximately 0.01 μl of blastocoel fluid (BF) was aspirated from each blastocysts using an ICSI pipette, which was inserted into the point of contact between two TE cells paying great attention to avoid the aspiration of any cell or debris. **B. Transfer blastocoel fluid into PCR tube. C. 2% agarose gel electrophoresis of WGA samples.** BF-blastocoel fluid, TE-trophectoderm biopsy and WB-whole blastocyst. Samples shown are from the same blastocyst.
(PDF)

S1 Table Results from optimization process evaluating WGA efficacy and downstream application in NGS PGT-A analysis using VeriSeq Kit of a combination of blastocyst conditioned culture medium (BCCM) and/or blastocoel fluid (BF). Several different approaches in collection of the samples were applied including: with/without d4 zona breaching (ZB), collection of the BF fluid with ICSI pipette together with BCCM.
(PDF)
