# Supplementary material for: Evaluation of a novel non-invasive preimplantation genetic screening approach
Source: PLoS One. 2018 May 10;13(5):e0197262. doi: 10.1371/journal.pone.0197262 (PMC5944986; doi:10.1371/journal.pone.0197262)
Supplement: S1 Fig — A. Blastocoel fluid (BF) collection. Aspiration of BF from a blastocyst using an ICSI pipette: gentle aspiration allows the blastocoel cavity to collapse. Approximately 0.01 μl of blastocoel fluid (BF) was aspirated from each blastocysts using an ICSI pipette, which was inserted into the point of contact between two TE cells paying great attention to avoid the aspiration of any cell or debris. B. Transfer blastocoel fluid into PCR tube. C. 2% agarose gel electrophoresis of WGA samples. BF-blastocoel fluid, TE-trophectoderm biopsy and WB-whole blastocyst. Samples shown are from the same blastocyst. (PDF) [file pone.0197262.s001.pdf]

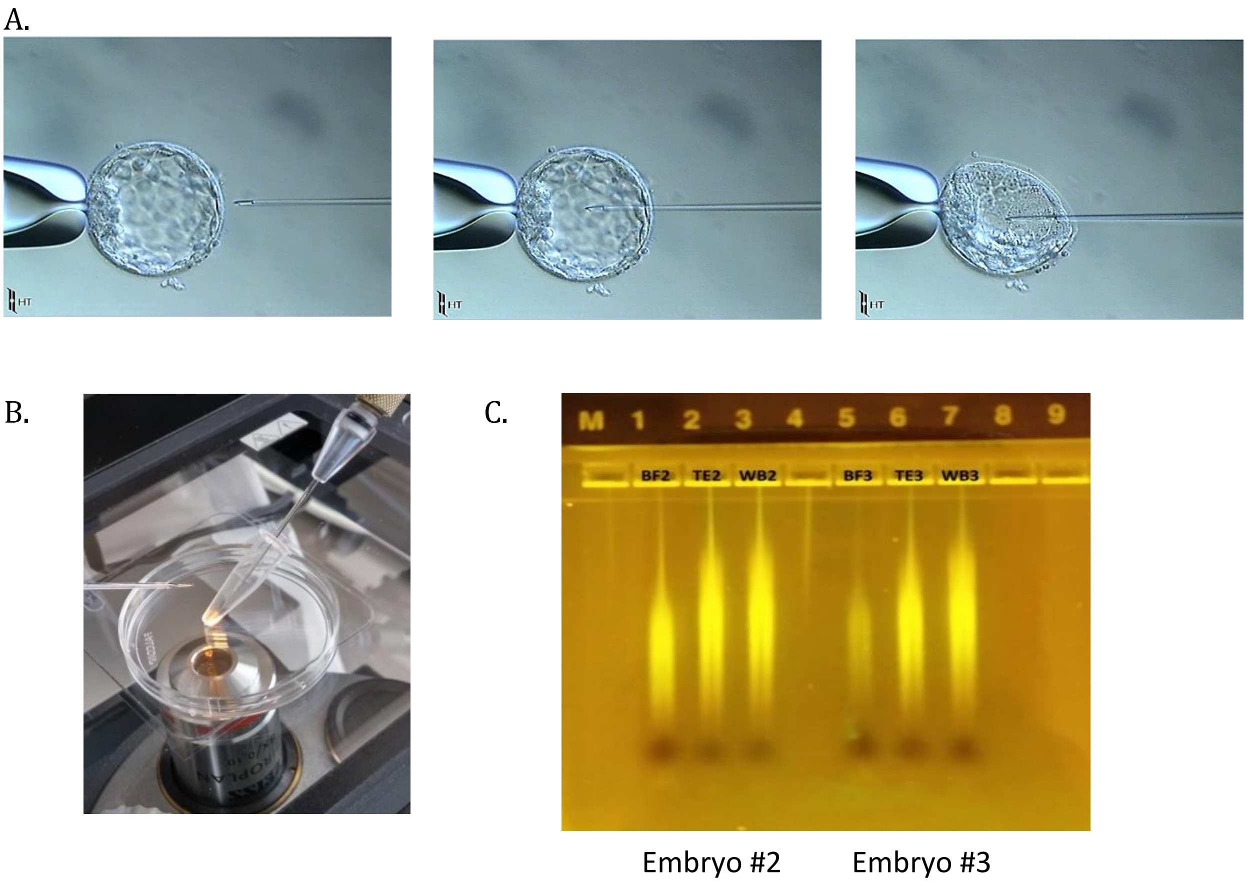

S1 Fig. A. Blastocoel fluid (BF) collection. Aspiration of BF from a blastocyst using an ICSI pipette: gentle aspiration allows the blastocoel cavity to collapse. Approximately 0.01 µl of blastocoel fluid (BF) was aspirated from each blastocysts using an ICSI pipette, which was inserted into the point of contact between two TE cells paying great attention to avoid the aspiration of any cell or debris. **B. Transfer blastocoel fluid into PCR tube. C. 2% agarose gel electrophoresis of WGA samples.** BF-blastocoel fluid, TE-trophectoderm biopsy and WB-whole blastocyst. Samples shown are from the same blastocyst.
