# Supplementary material for: Evaluation of a novel non-invasive preimplantation genetic screening approach
Source: PLoS One. 2018 May 10;13(5):e0197262. doi: 10.1371/journal.pone.0197262 (PMC5944986; doi:10.1371/journal.pone.0197262)
Supplement: S1 Table — Several different approaches in collection of the samples were applied including: with/without d4 zona breaching (ZB), collection of the BF fluid with ICSI pipette together with BCCM. (PDF) [file pone.0197262.s002.pdf]

Supplement Table 1. Results from optimization process evaluating WGA efficacy and downstream application in NGS analysis using Veriseq Kit of a combination of blastocyst conditioned culture medium (BCCM) and/or blastocoel fluid (BF). Several different approaches in collection of the samples were applied including: with/without d4 zona breaching (ZB), collection of the BF fluid with ICSI pipette together with BCCM.

Patient number	Sample type	WGA (SurePlex)-DNA concentration (ng/µl)	NGS results
1	BCCM+BF	14.8	Clinical grade QC
2	BCCM+BF	12.2	Clinical grade QC
3	BCCM+BF	17.6	Clinical grade QC
4	BCCM+BF	5.1	No result
5	BCCM+BF	11.8	Clinical grade QC
6	BCCM+BF	11.8	Interpretable/noisy
7	BCCM+BF	16.6	Clinical grade QC
8	BCCM+BF	14.0	Clinical grade QC
9	BCCM+BF	22.9	Clinical grade QC
10	BCCM+BF	11.9	Interpretable/noisy
11	BCCM+BF	13.8	Clinical grade QC
12	BCCM+BF	14.4	Clinical grade QC
13	BCCM+BF	8.4	Interpretable/noisy
14	BCCM+BF	7.9	Interpretable/noisy
15	BCCM	12.2	No result
16	BCCM	11.6	No result
17	BCCM	16.8	Interpretable/noisy
18	BCCM	12.4	Interpretable/noisy
19	BCCM	9.92	No result
20	BCCM	15.3	Interpretable/noisy
21	BCCM	7.2	No result
22	BCCM	22.6	Interpretable/noisy
23	BCCM	9.9	No result
